# Universal Interatomic Potentials with DFT for Understanding Orbital Localization in Polydimethylsiloxane-Amorphous Silica Nanocomposites

**DOI:** 10.1021/acsomega.5c09273

**Published:** 2025-12-05

**Authors:** Carson Farmer, Hector Medina

**Affiliations:** School of Engineering, 5199 Liberty University, 1971 University Blvd, Lynchburg, Virginia 24515, United States

## Abstract

To investigate molecular orbital localization in polydimethylsiloxane (PDMS) amorphous silica nanocomposites, an approach that integrates first-principles with data-driven methods is introduced: Integrated Modeling and Prediction using *Ab initio* and Combined Trained potentials for orbital localization (IMPACT4OL). This approach is used to accelerate the identification of localized orbitals near the polymer–nanoparticle interface in polymer nanocomposites (PNC). To accelerate the structure generation, machine-learned interatomic potentials (MLIPs) are utilized to perform molecular dynamics studies for studying the interfacial region with quantum mechanical methods. To investigate the adsorption behavior, various cross-linking densities of PDMS are utilized. While cross-links have been shown to induce defect sites for deep traps, for a small degree of polymerization of PDMS, they hinder the surface adsorption and, thereby, the locations of localized orbitals, which, in turn, can serve as trap sites. The acceleration of orbital localization, especially in large model systems, using IMPACT4OL facilitates the elucidation of intricate mechanisms and could help advance the development of novel PNC-based insulators and electrets. Furthermore, our method introduces a new path to advance understanding of the conformational dynamics for polymer–nanoparticle interface science and engineering.

## Graphical abstract

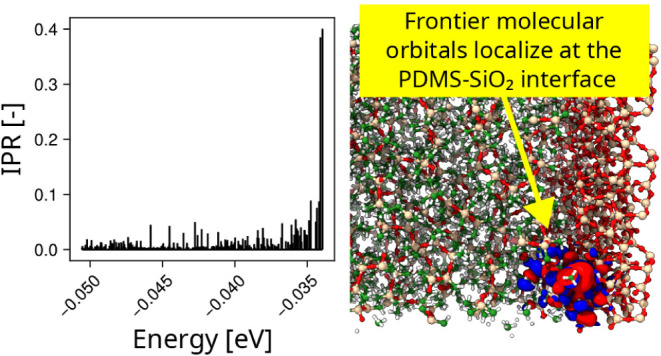

## 1 Introduction

The bulk properties of polymer nanocomposites are partly governed by the interactions between the polymer and nanofiller and the interfacial region formed. The interfacial region can be used to tune both mechanical properties, such as glass transition temperature,^1,2^ and electrical properties, such as dielectric permittivity^3,4^ and electrical breakdown strength.^5^ Furthermore, the effects of the interfacial region extend further into the quantum mechanical (QM) regime where localization of molecular orbitals,^6^ dielectric permittivity differences,^7^ and charge carrier transport^8^ can all potentially arise. Combining these different features of polymer nanocomposites heralds the development of improved electrical insulators^9–11^ and electrets,^12^ including dielectric elastomers,^13–15^ and other applications.^16^ Further, improved electrets have applications in improving displacement sensing,^17^ actuation,^18^ and energy generation.^19^

The properties of the interfacial region in polymer nanocomposites is, in part, governed by particle–polymer interactions^20,21^ and polymer chain length.^22^ Further, external effects, such as applied electric fields,^7^ can produce changes in the structure and orientation of the polymer chains at the interface. However, capturing these dynamics has traditionally required the use of quantum mechanical (QM) methods such as density functional theory (DFT). The use of QM methods enables researchers to probe the trap properties for simple polymer systems.^23–25^ Previous research examining charge localization effects has been constrained to investigations of either polymer-small slab interactions^6^ or systems with a limited number of polymer chains (fewer than 5).^25,26^ Large system sizes have previously been intractable with DFT methods scaling with $O\left(n^{3}\right)$ - $O\left(n^{6}\right)$ depending on the level of theory. For example, Saiz and Quirke reported times of greater than 10 h for 1 ps of molecular dynamics with CP2K on 64 cores. Recently, advances in machine-learned interatomic potentials (MLIPs) have provided models which scale with O(n)^27–29^ and are applicable to a wide range of chemical systems outside of their training domain.^30^ These advances are enabling accelerated rates in the discovery of materials,^31^ capturing the dynamics of larger systems,^32^ and using quantum methods, where appropriate, to find the origins of new phenomena.^33^ Of particular interest are the nonconservative or direct predictions developed in the Orb models,^27,34^ which have been demonstrated to learn physical information and are capable of being evaluated on out-of-distribution structures.^30^

The majority of the studies on polymer trap systems have focused on polyethylene (PE) with various additives.^35–37^ These systems are a common choice for electrical insulation and have been shown to exhibit charge trapping phenomena. However, limited research has been conducted on charge trapping in PDMS-amorphous silica (aSiO_2_) nanocomposites.^38–40^ Additionally, limited studies have been conducted on the influence of PDMS defects/modifications on the trapping phenomena.^41^ Experimental results from Zhang et al.^17^ have demonstrated that cross-linked PDMS-aSiO_2_ can be used to create stable and long-lasting stretchable polymer electrets. However, their work provides no evidence of the mechanisms by which this is accomplished. Recently, experimental results from^38,42^ demonstrated that PDMS (Sylgard 184) with silica particles (30 nm diameter) developed space charges when exposed to prolonged electric fields (>10 kV/mm for 2 h). The limited work on PDMS-aSiO_2_ has prevented a direct connection to charge trapping and orbital localization in the materials. In the absence of experimental data for measured density of states, computational simulations can provide insight into the localization and trapping phenomena that occur in the material.

Inspired by our previous integrated method,^43^ this work develops a method for Integrated Modeling and Prediction with *Ab initio* Combined with Trained Potential for Orbital Localization (IMPACT4OL), and it is implemented to uncover the mechanisms of molecular orbital localization in PDMS-aSiO_2_ as a proof of concept (see Figure 1). Using Orb-D3-V2 ^34^ as the MLIP for the study, modeled PDMS-aSiO_2_systems are produced with molecular dynamics (MD) and fully equilibrated with time scales on the order of picoseconds (ps), which otherwise would be intractable with traditional QM methods, and interfacial regions large enough to capture the polymer chain interactions. Then with DFT calculations, the inverse participation ratio (IPR) is used to probe the localization induced by the polymer-surface interface.

**Figure 1:**
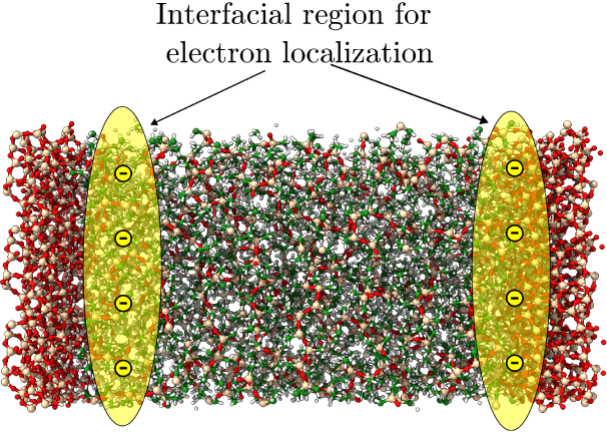
For the PNC system of PDMS-aSiO_2_, the orbitals are shown to localize at the interfacial region and produce trap states for carriers at the interface between the two materials. The model system is shown with periodic boundary conditions. The PDMS system is placed between amorphous SiO_2_ slabs and equalized.

## 2 Methods

The process of studying the localization of electrons in the systems is divided into three steps: 1) initial generation of PDMS and SiO_2_ structure, 2) MD of the total system, and 3) QM analysis of the MD snapshot for localization metrics. For the initial structure generation MD steps, MLIPs are utilized to accelerate the structure generation process. All MD simulations were performed with ASE 3.24 and a time step of 1 femtosecond (fs), unless otherwise stated.

### 2.1 Generation of Initial Structures

To generate the amorphous silica (aSiO_2_) slabs, an initial structure of beta-cristobalite from the Materials Project^44^ (mpid: 546794) is formed into a 3 × 3 × 3 supercell. Orb-D3-V2MLIP^34^ was used to calculate the forces and energies for the MD steps required to generate the amorphous structure. While recent Orb-V3 models are available, the selection of a model trained with D3-dispersion interactions was selected to capture the long-range intermolecular interactions that occur in the polymer-slab interface.^45,46^ Without additional benchmarks on Orb-V3 for intermolecular interactions, Orb-D3-V2 was chosen since the trained data had D3 corrections augmented, whereas Orb-V3 was trained without D3 corrections. The system was equilibrated under the NVT ensemble at 300 K with the Bussi thermostat^47^ and subsequently under the NPT ensemble at 300 K and 1 atm with the Berendsen barostat and thermostat.^48^ Then, a melt-quench process is performed with a melting rate of 300 K/ps. The system was heated to a maximum temperature of 6000 K and held at that temperature until density fluctuations were less than 1% in the NPT ensemble at 1 atm. The system was then quenched at a rate of 600 K/ps to 300 K. Next, the unit cell was cleaved in the *z*-direction. Silicon atoms with coordination numbers of 1 or 2 were removed. Silicon atoms with a coordination number of 3 had an oxygen added to complete a tetrahedron with the three existing bonded oxygen atoms. Lastly, hydrogen atoms were added to all 1-coordinated oxygen atoms. The surface had a vacuum gap of 2 nm added, and the system was equilibrated at 300 K in the NPT ensemble. The final slab structure was tiled in a 3 × 3 pattern in the *x*- and *y*-directions for the slab used in the latter steps.

Now, we describe the methods used to generate the PDMS models. For each degree of polymerization (DP) studied, a model PDMS chain of 12 monomers long was generated using the mBuild^49^ library. The chain was subsequently optimized in a unit cell 10 times greater than the largest dimension of the PDMS. The chain was optimized with Orb-D3-V2.^34^ Based on Popov et al.,^50^ short chains were selected to create a shallow interfacial region. The shallow region prevents the interfacial dynamics from extending across the periodic boundary conditions. Next, the chains were packed into a box with a cross-section equivalent to the final slab cross-section and a density of 0.2 g/cm^3^. The system was equalized under the NVT ensemble at 300 K for 1 ps and then under an inhomogeneous NPT (*z*-only) ensemble at 300 K and 1 atm until density fluctuations were less than 1% over a 3 ps sample.

To generate cross-linked structures, the force field and cross-linking process from Khot et al.^51^ were utilized to generate a cross-linked PDMS network similar to Sylgard 184, which exhibits a hydrosilylation reaction to form cross-links between the end groups of part A chains and select monomers in part B chains (see Figure 2). The cross-linking was performed at 300 K and 1 atm in the NPT ensemble. Bonds were formed if reactive beads were within 3 times the relaxed C–C bond distance. After the bond was formed, a geometry optimization of the system occurred before resuming the MD simulation. Bond formation occurred every 5 ps until the desired cross-linking density occurred. Hydrogens were added back to the system, and a similar NVT/NPT ensemble was used to equalize the system. Both part A and part B chains were 12 monomers long.

**Figure 2:**
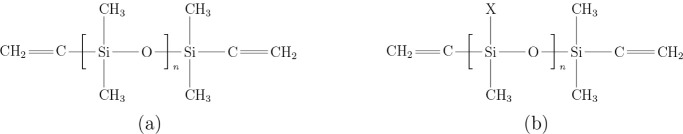
To simulate the hydrosilylation reaction used for cross-linking Sylgard 184, chains from (a) are modeled having *n* monomers with reactive end groups, and chains from (b) have the same total number of monomers; however, the placement of the reactive groups is randomly selected along the backbone with a 50% probability of the unit having a hydrogen (H) atom compared to a methyl group, and the group is placed at the “X” location.

### 2.2 Molecular Dynamics Overview and Charge Trapping Evaluation

The PDMS was unwrapped in the *z*-direction and placed with a 2 Å gap above the SiO_2_ slab. The system was equalized in the NVT ensemble for 5 ps and then compressed under an inhomogeneous NPT ensemble in the thickness direction until density fluctuations of less than 1% were observed during a 5 ps sample.

Next, the final snapshot from the MD simulation is used for charge characterization. The snapshot is optimized in CP2K 2025.1 ^52^ first with GFN1-xTB for 25 steps. The GFN1-xTB optimization was found to aid in the convergence of the subsequent DFT calculations. Then, the Quickstep method using the GTH-PBE potential,^53^ DZVP-MOLOPT-PBE-GTH basis set,^54^ and the PBE functional with the GPW method in the Quickstep module of CP2K. The 200 orbitals adjacent to HOMO and LUMO were used in the localization analysis. To analyze the localization of the orbitals at the PDMS-SiO_2_ interface, the inverse participation ratio (IPR) was utilized:^55,56^

1 $$IPR_{i}=\int_{\Omega }|\Psi_{i}\left(r\right){|}^{4}dr$$

where Ψ_*i*_ represents molecular orbital *i*. In practice, eq 1 is discretized by finite volumes for evaluation from a cube file:

2 $$IPR_{i}\approx \underset{j}{\sum }\Psi_{i}{\left(r_{j}\right)}^{4}\Delta V$$

where Ψ_i_(*r*_j_) is the value of the molecular orbital from the cube file at sampled point *r*_j_ and Δ*V* is the unit volume from the cube file. The cubefiles are generated using a CP2K stride of 1.

## 3 Results

First, we validated the aSiO_2_model. Based on the melt-quench process for aSiO_2_, the density was found to converge to 2.22 g/cm^3^, which is only about 1% different from the experimental density.^57^ Based on this, the Orb-D3-V2 model is applied to model the structure and dynamics of the aSiO_2_ surface and additionally the PDMS bulk material. The final unit cell and density convergence are shown in Figure 3. Extending the models of the prior works of Shandilya et al.^26^ and Saiz and Quirke^6^ for modeling aSiO_2_ and polyethylene interfaces, the model of aSiO_2_ is used as a slab for placing a series of PDMS chains and cross-linked units between. An example of the complete system is shown in the inset of Figure 3.

**Figure 3:**
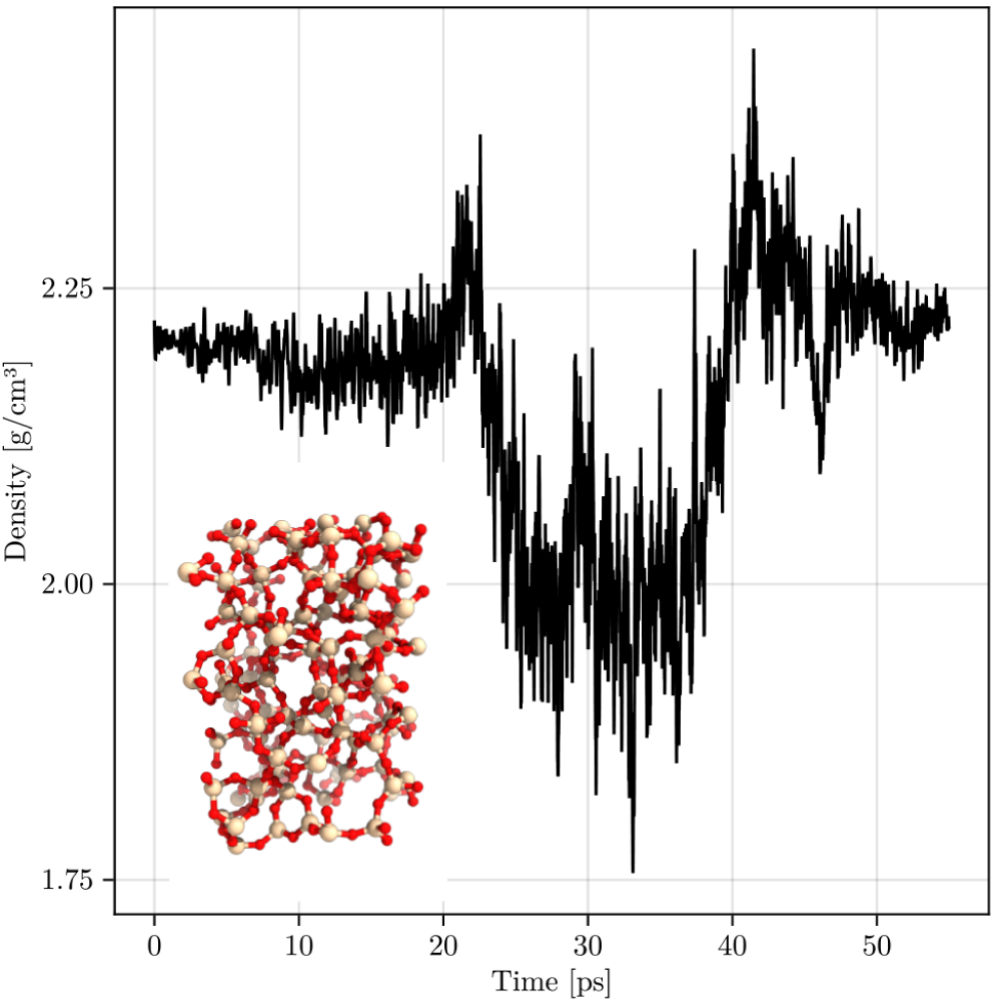
Initially, the sample is warmed to 300 K and is then heated to 6000 K, equilibrated, and quenched. The final structure is shown as an inset. After the melt-quench process is completed, the final cell density is 2.22 g/cm^3^.

After equilibrating the PDMS-silica system and a subsequent geometry optimization, the PDMS-SiO_2_ systems were analyzed in CP2K to understand the electron localization effects. The IPR is calculated for 200 orbitals from HOMO and 200 orbitals from LUMO. The results are shown in Figure 4. The orbitals are shown at the corresponding energy levels. The increase in cross-linking sites produces an increase in localization locations. For reference of the electron localization, an IPR of 0.0073 would correspond to localization over approximately 137 atoms or one chain of PDMS consisting of 12 monomers.

**Figure 4:**
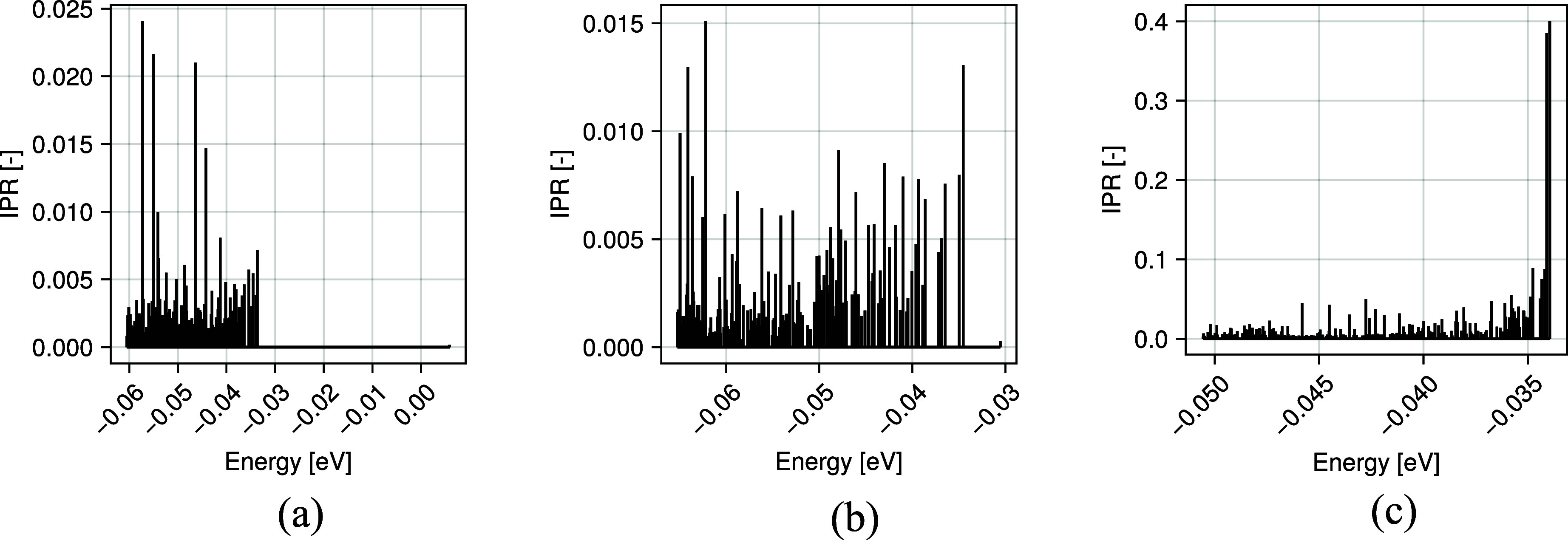
(a) 0 cross-links, (b) 23 cross-links, (c) 55 cross-links. With increasing cross-linking, the density of localized states in the systems increases near the band edge.

To further inspect the regions near the band edges where the orbitals become highly localized, ±0.001 isosurfaces of the molecular orbital are visualized. The IPR is calculated based on eq 2. Shown in Figure 5 are two orbitals, where in Figure 5a, the orbital is localized to the PDMS-aSiO_2_ interface and has an IPR of approximately 0.4 compared to a delocalized orbital shown in Figure 5b. The delocalized orbital tends to be spread over a large area and across multiple chains. However, for the localized orbital, the electrons tend to localize over a region of a polymer chain near the interface and in a region that is interacting with the aSiO_2_surface.

**Figure 5:**
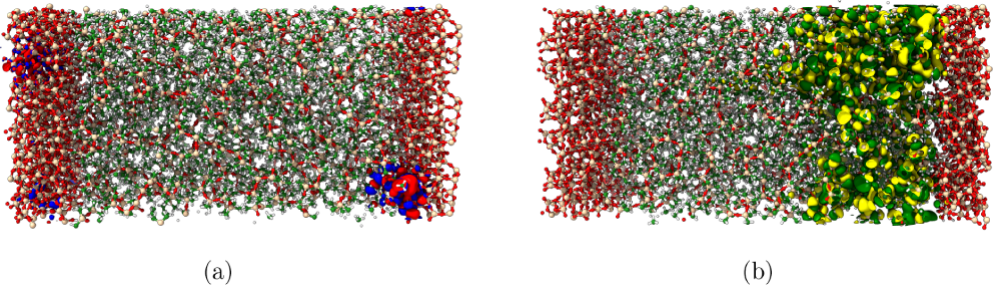
(a) For the highly localized state at the occupied band edge, the 0.001 e/bohr^3^ isosurface is localized to the interfacial region between the PDMS and SiO_2_ compared to the orbital with a localization of 0.003 (b), where the orbital is delocalized over a region of the PDMS.

## 4 Discussion

### 4.1 MLIP Verification for PDMS-Silica

The effectiveness of IMPACT4OL in accomplishing the task of accelerating the prediction of orbital localization for larger and complex systems lies in its main components: MLIPs were combined with (*ab initio*) methods. The application of the Orb-D3-V2 network for modeling the formation of aSiO_2_ highlights the potential for MLIPs trained on primarily crystalline material samples to capture high-temperature melting dynamics and then the quenching process for forming aSiO_2_. While traditional force field approaches are known to be capable of creating aSiO_2_ models, the use of a generalizable MLIP to capture the densification and melt-quench process for aSiO_2_ highlights the generalizability to out-of-distribution systems. Because Orb-D3-V2 was trained on MPTraj^58^ and Alexandria,^59^ the training data did not include amorphous structures similar to those found in both the aSiO_2_ and some PDMS regions. The forces learned as a result of varying from ground-state to higher-energy-state crystalline structures led to successfully forming aSiO_2_ unit cells with densities converging to reported experimental density and structures similar to other reported aSiO_2_ cells. From the MD results on the system, the model was able to capture the correct forces and dynamics without knowledge of similar structures. The results of modeling the PDMS-aSiO_2_ system seem to indicate that MLIPs trained on a diverse data set are capable of reproducing polymer system dynamics. Finally, even though we suspect that the addition of the D3-dispersion term to the training data led to an improved conformation of the PDMS-aSiO_2_ interface, validating parameters are yet to be provided.

### 4.2 Orbital Localization in PDMS-Silica

From the localization study, the IPRs for the different structures are shown in Figure 4. For the sample without cross-linking, the higher IPRs are found to be caused by chain rearrangement toward the surface of aSiO_2_. Based on,^60^ the chains are expected to reorient toward the surface of the silica. This is validated using the density along the thickness of the specimen (Figure 6). As the aSiO_2_ attacts the PDMS chains, causing local chain reorientation, the samples without cross-linking exhibited higher localization compared to the cross-linked counterparts. However, the interface of the cross-linked sites tends to exhibit a mean IPR near the band edge of 0.07, which is at a level that compares to the localization in a single PDMS chain.

**Figure 6:**
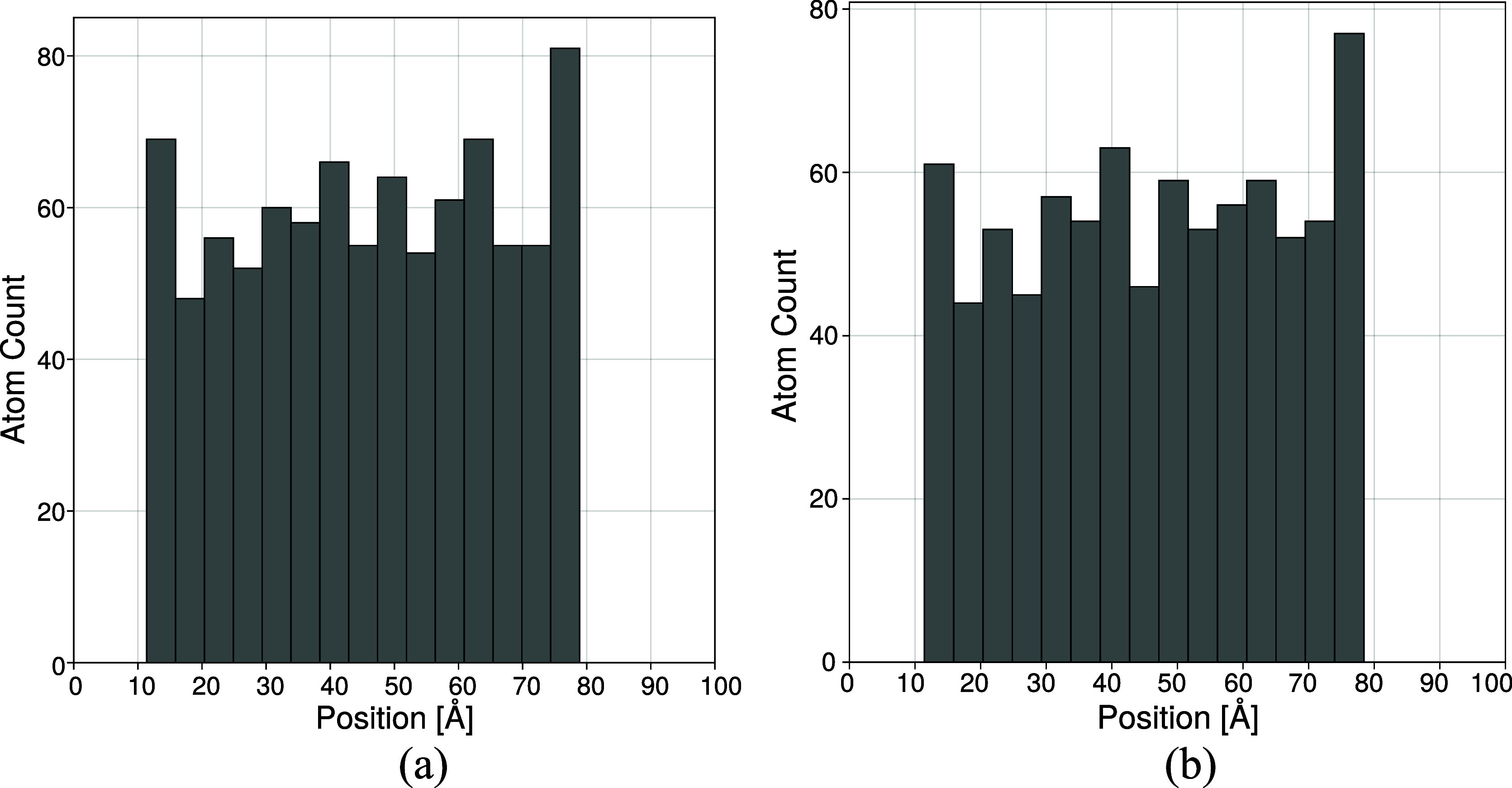
PDMS chains rearrange to adhere to the bulk silica surface, as shown by the peaks in the Si (a) and O (b) atomic density profiles near the interfacial regions.

Furthermore, comparing the 55-cross-link structure’s localized orbital against a delocalized orbital provides insight into the mechanism for localization formation at the interface. For the orbitals with high IPR, the orbital localized to a region where the end methyl group of the PDMS was able to interact with a void at the interface with aSiO_2_ (see Figure 7). The interaction suggests that the surface (or face) characteristics of aSiO_2_ may contribute to the localization interactions at the interface. While further studies are required to understand the implications of aSiO_2_ surface characteristics and PDMS interactions, this initial mechanism suggests that nanoparticles with pores and surface voids at the scale of the methyl group may lead to enhanced localization and thereby charge trapping. One practical design choice would be to use fumed silica particles which are particles with large surface voids/pores for enhancing the number of highly localized orbitals in the material.^61^

**Figure 7:**
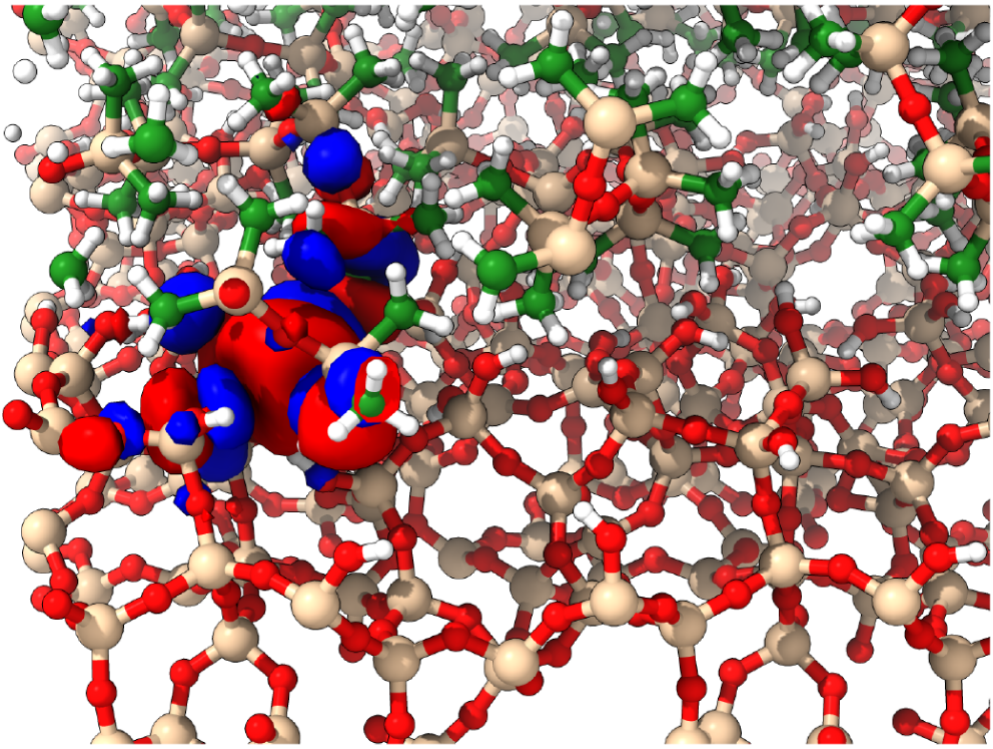
Localization of the orbital with an IPR of 0.4 in the void of the silica surface interacting with the end group of the PDMS chain. Green atoms represent carbon; white atoms represent hydrogen; red atoms represent oxygen; and beige represents silica.

## 5 Conclusions

The successful and accelerated modeling of the orbital localization at the PDMS-aSiO_2_ interface highlights the potential for trap engineering to help advance the development of both insulating devices and electrets, at least. The acceleration of structure generation via MLIPs provides a new approach to understand the conformational dynamics for polymer–nanoparticle interface engineering,^62–66^ which is a topic of high importance in materials science. While future research is needed to understand the current limitations of MLIPs for a broad range of PNC systems, the systematic verification of the model for PDMS-aSiO_2_ provides the gateway to develop robust methods to sample the conformational changes in PNC systems, where accurate force fields have not been developed. While this study focused on snapshots and short-time scale molecular dynamics, the investigation of longer time scales and larger systems may reveal key dynamics of the polymer chains near the nanoparticle surface. Additionally, other sources of localization may be induced by the presence of defects or other impurities in the polymer matrix, a state of affairs that would require further studies. Localization occurs in regions where the PDMS chains have more freedom to reorient to the aSiO_2_ surface. Lastly, additional experimental research on the PDMS-aSiO_2_ system, such as measured density of states, dielectric breakdown strength, and trap levels, is required to fully validate the findings in this work. Future research on the potential defect sites in the PDMS network structure may give rise to new features that should be included for electron localization at the PNC interface. IMPACT4OL outlines the approach of combining MLIPs with *ab initio* methods for studying localization effects in the example of polymer nanocomposites. As new MLIP models are developed, the system sizes and chemistries that can be explored will continue to grow. The proposed workflow could be used for generalization of localization effects in a range of polymer–filler systems and provide guided insights into better engineering of dielectrics and interfacial interactions.
